# Affordable Artificial Intelligence-Assisted Machine Supervision System for the Small and Medium-Sized Manufacturers

**DOI:** 10.3390/s22166246

**Published:** 2022-08-19

**Authors:** Chen Li, Shijie Bian, Tongzi Wu, Richard P. Donovan, Bingbing Li

**Affiliations:** 1 Autonomy Research Center for STEAHM (ARCS), California State University Northridge, Northridge, CA 91324, USA; 2 Department of Computer Science, Columbia University, New York, NY 10027, USA; 3 Language Technologies Institute, School of Computer Science, Carnegie Mellon University, Pittsburgh, PA 15213, USA; 4 California Institute for Telecommunications and Information Technology (Calit2), University of California Irvine, Irvine, CA 92297, USA; 5 Department of Manufacturing Systems Engineering and Management, California State University Northridge, Northridge, CA 91330, USA

**Keywords:** affordable AI accelerator, computer vision, smart manufacturing, machine supervision, 3D printing, IoT

## Abstract

With the rapid concurrent advance of artificial intelligence (AI) and Internet of Things (IoT) technology, manufacturing environments are being upgraded or equipped with a smart and connected infrastructure that empowers workers and supervisors to optimize manufacturing workflow and processes for improved energy efficiency, equipment reliability, quality, safety, and productivity. This challenges capital cost and complexity for many small and medium-sized manufacturers (SMMs) who heavily rely on people to supervise manufacturing processes and facilities. This research aims to create an affordable, scalable, accessible, and portable (ASAP) solution to automate the supervision of manufacturing processes. The proposed approach seeks to reduce the cost and complexity of smart manufacturing deployment for SMMs through the deployment of consumer-grade electronics and a novel AI development methodology. The proposed system, AI-assisted Machine Supervision (AIMS), provides SMMs with two major subsystems: direct machine monitoring (DMM) and human-machine interaction monitoring (HIM). The AIMS system was evaluated and validated with a case study in 3D printing through the affordable AI accelerator solution of the vision processing unit (VPU).

## 1. Introduction

### 1.1. Background

The incredible pace of deployment of AI technology is driving the sudden emergence of the fourth industrial revolution (Industry 4.0). The rapid evolution of this manufacturing paradigm is driving an urgent need to create useful tools for designing and deploying diverse data models. Small and medium-sized manufacturers (SMMs) typically lack the appropriate resources to affordably establish the IT infrastructure needed for gaining visibility of factory operations (workflow and materials use) and real-time machine monitoring which is the promise of Industry 4.0. Using consumer visualization tools developed for smartphones, smart home applications, and utility smart meters installed by utilities will ease information technology (IT) infrastructures’ demands for acquiring data to assess workflow and energy/materials usage. By classifying workers’ activities and traffic trajectory inside the facility, the states of equipment usage and the workflow can be acquired. A system identifying the correlation between the states of equipment/workflow enables workers and supervisors to gain insight into the operational footprint. The continuous streaming machine status and manufacturing operation insight can optimise factory operation for production efficiency and dynamic scheduling of equipment used to lower energy costs in multi-tier electricity supply markets. This coordinated data acquisition system from visualization and state sensors is a critical IT infrastructure for smart manufacturing.

The coordinate data acquisition system is especially crucial for the affordable, scalable, accessible, and portable (ASAP) solution of smart connected worker advanced manufacturing systems. For the AI embedded in these systems to generalize to a broad context, these systems will need to be as diverse in their architectures as the manufacturing ecosystems they will operate. These manufacturing ecosystems are computationally bound through a network of manufacturing activities, tools, machinery, and inputs (i.e., materials, resources, energy) configured through data-enabled manufacturing processes and enacted by workers augmented with additional intelligence to perform adaptive manufacturing tasks. The challenge is to develop design tools that will enable engineers to reliably assess and implement data flow requirements for ASAP smart connected workers across all manufacturing sectors while focusing on computer vision-based approaches for improving productivity, automated demand-side management, detecting errors, minimizing waste streams, and improving sustainability performance.

To use reliable methods to automate these supervisory tasks, this research proposes an Artificial Intelligence-assisted Machine Supervision (AIMS) system, which tracks the working state of the machines through real-time observation of the machine. The AIMS does not rely on any data transmitted directly from the machine to determine the machine’s status. If the machine’s control system fails to detect an abnormal condition, the AIMS can detect the abnormality by observing the machine. When building an AIMS on a particular machine, the designer must thoroughly understand the machine’s possible standard workflow in the context of human interactions. In practice, the AIMS will immediately detect anomalies when the machine components go beyond the range specified by the standard workflow.

### 1.2. Related Works

Recent works have proposed to advance the processes of industrial production and supervision. Bian et al. proposed and developed a centralized and automated real-time monitoring system, utilizing numerous machine learning-based techniques to reduce the cost of human labor and improve energy efficiency in smart manufacturing [1]. Peres et al. deployed a framework called Intelligent Data Analysis and Real-Time Supervision for the manufacturing environment [2]. Based on RFID-tagged intelligent electric energy meters, a production and manufacturing supervision management system was developed and tested [3]. Using the IoT techniques, Tiboni et al. presented a smart module architecture and obtained efficient supervision and monitoring in an industrial setting [4]. Lapidus and Topchiy formed a series of control measures for construction supervision at the facility and in the office [5]. The technological breakthroughs brought about by the rapid development of machine learning-based computer vision [6] in recent years have provided a solution worth exploring for the automation of industrial production supervision. M.R. Valero et al. [7] have designed a coherent framework for smart manufacturing. A concept has been proposed for supporting the collaborative practice platforms in smart manufacturing by Luís Ferreira et al. [8] Using various sensors, an Edge-to-Cloud Industry Internet of Things framework has been developed for condition monitoring in manufacturing systems by Zhi Li et al. [9]. In addition, object detection and text recognition are two promising branches of computer vision that can potentially push automated supervision to a new level.

**Object detection** has seen increasing application in various fields, most recently in smart manufacturing. Wiech et al. applied an optical object detection system in a learning factory setting to detect human errors [10]. Overcoming the self-occlusion for texture-less objects, Cong et al. proposed an online 3D object detection model and an effective estimation method afterwards [11]. Lemos et al. used deep learning-based object detection techniques to enhance the adaptability and effectiveness of robotic manipulators for parts identification in additive manufacturing [12]. Leveraging the processing speed of object detection models, Bian et al. developed a real-time system that could monitor machine states and perform automatic fault detection on 3D printers [13].

**Text recognition** is also rapidly developing due to the advancement of various AI techniques. Wang et al. addressed some challenges of text detection in the industry through an algorithm called Progressive Scale Expansion Network, which resulted in high-performance accuracy [14]. Yu et al. took another approach and focused on the semantic information of text and developed a novel Semantic Reasoning Network which outperformed the previously used Recurrent Neural Network (RNN) method [15]. Based on the commonly used segmentation method for scene text detection, Liao et al. performed a binarization process in the segmentation, which achieved good performance [16]. Hu et al. used the guided training of Connectionist Temporal Classification and obtained a model with high prediction accuracy and robustness [17].

### 1.3. Contribution

The developed AIMS system follows the same logic as traditional supervision methods and can improve the effectiveness of human supervisors in manufacturing scenarios, thus improving productivity and reducing labor costs. Specifically, we summarize the contribution of this work as the following items:

- A novel method for anomaly detection and state change detection based on the AI-assisted method;
- To significantly reduce the computational difficulties while clarifying logic and reducing system maintenance difficulties;
- Deployed cloud computing technology and developed the system on embedded devices to make it affordable for SMMs to implement the AI-based Smart Manufacturing practices;
- Incorporated the human-computer interaction monitoring to ensure that the corresponding action has an expected impact on the machine state, which has safety and cybersecurity implications.

## 2. Framework

**Figure 1:**
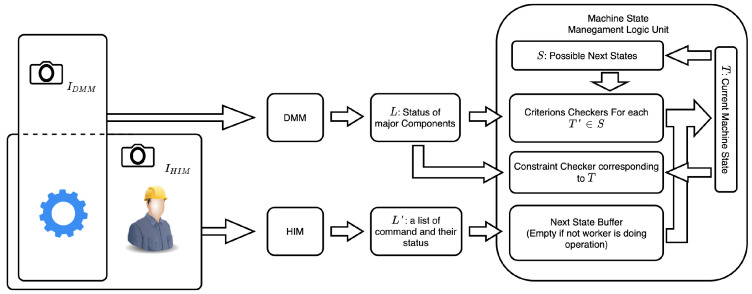
The framework of AIMS.

The proposed AIMS system consists of two major subsystems: direct machine monitoring (DMM), and human-machine interaction monitoring (HIM), as shown in Figure 1.

### 2.1. Machines Applicable to AIMS

The AIMS applies to any manufacturing environment but is particularly relevant in scenarios with manual operations or facilities with fewer automation features that need real-time monitoring or supervision (e.g., many SMMs.) In actual production, it is required to supervise the machine’s working state at all times. When a specific machine is identified for the application of the AIMS, an analysis of how the machine works are required before designing an AI-supervised model to achieve more accurate and efficient supervision. The following analysis should be performed for a specific supervised device in the AIMS pipeline.

#### 2.1.1. Identification of Major Working Components

For most manufacturing equipment, it is not easy to understand the operating conditions from direct observation of the entire machine. AIMS utilizes the real-time monitoring of the motion of the major components (e.g., moving parts like tool changers or extruder heads) inside the machine to analyze the operating conditions of the entire machine. While this paper is explicitly focused on motion in the sense of state changes in physical space, the approach can also be applied to a more generalized concept of motion which can be extended to radiating systems like heaters and radios, as discussed in future work. This component-based identification provides a more precise observation accuracy than direct observation of the entire machine. It is also an accurate way to analyze the machine’s status based on the internal structure. The following section explains how the implementation of this approach uses artificial intelligence-based objection detection.

#### 2.1.2. Machine State Transition Diagram

Building the AIMS for a particular class of machines requires the definition of the state space, which includes the operating states of the overall machine, operating states of the major supervised components, and the constraints associated with prescribed operational parameters (e.g., speed, voltage, temperature, etc.) The machine state transition diagram graphically represents operational states and these associated constraints. Individual machines within a particular class will likely exhibit some variation from the class-based state diagram addressed in AIMS through the implementation of adaptive AI training. After confirming the current state, the constraints on the primary component corresponding to the current state are analyzed. Once our supervised model detects broken constraints, the system can recognize a machine’s operational envelope problem. At the same time, AIMS makes a state diagram of the transition relationships of each state. The supervised model can obtain a set of possible subsequent states from the state diagram based on the current state. For each possible subsequent state, the model can check the motion state of each major component to confirm whether the threshold to enter the state is satisfied. In our experiments, a boolean First-in First-out queue of capacity five is applied to record the thresholds of the last five frames for each possible next state. If 80% of the thresholds in the last five frames are satisfied, the machine is determined to enter the next state corresponding to the queue. The use of state diagrams dramatically reduces computational complexity while improving accuracy. Because the AIMS model understands the state diagram, at any point in time, the model can call the validation function to determine whether each major component is in a legitimate state based on the state diagram instead of calling a tedious general error checker, which can be error-prone and computationally prohibitive. Moreover, the use of state diagrams also dramatically reduces the computational effort of state transformation. Without state diagrams, a model would need to analyze the motion states of the major component of multiple consecutive frames in the past and analyze each possible combination of future states. Such an analysis is highly complex. With the help of state diagrams, inputting the current state helps the model invoke the exact threshold that needs to be checked. This reduces the amount of computation, streamlines the logic, and significantly reduces the possibility of incorrect codes and the difficulty of maintaining the code.

#### 2.1.3. Working Condition Analysis

For the system to have more accurate predictions, the working environment of the manufacturing equipment also needs to be considered. Of particular note are those conditions that can affect the computer vision model. With the understanding of the working condition, a more robust model can be trained.

### 2.2. Direct Machine Monitoring (DMM)

The Direct Machine Monitor (DMM) utilizes computer vision to supervise the state of motion of the main components of a manufacturing machine. AIMS uses a general camera to capture images of the interior of the supervised machine in real-time. Each image $I_{DMM}\in R^{3\times W\times H}$ is fed to the computer vision model in an RGB three-channel tensor structure. After a convolutional computation, the computer vision model generates a list *L*. The length of *L* is the number of objects recognized in the image. $L_{i}$, the *i*-th element of the list, corresponds to the *i*-th object identified from $I_{DMM}$ and contains information about the type of the *i*-th object and its position in pixel coordinates on the image. If there are *n* major components that AIMS needs to track, then the type is an integer in 0,1,⋯,n−1 to indicate which major component the current entry of *L* is describing.

For any moment in the machine’s operation, the camera inside the machine captures a picture of its interior $I_{DMM}$. Then $I_{DMM}$ is fed to the DMM, and a list *L* is generated describing the location states of the major components inside the machine at the current moment, after which the DMM retrieves the set *S* of possible states corresponding to the current state *T* of the machine during the process. Then for all $T^{\prime }\in S$ ($T^{\prime }$ standards for an arbitrary possible next state), DMM checks if *L* satisfies the transition threshold of $T-T^{\prime }$ ($T-T^{\prime }$ stands for the transition from state *T* to state $T^{\prime }$) and adds the result to the queue corresponding to $T-T^{\prime }$ transition. If all the checks for transition fail, the program will pull out the error checker corresponding to the current state. After all these computations, the program will enter the next operation frame.

### 2.3. Human-Machine Interaction Monitoring (HIM)

To get a more accurate assessment of the working state of the machine, AIMS uses another model to detect the human-machine interaction. This system observes the machine’s human operation from outside and calls the corresponding operation’s handler function to join the machine state’s logic judgment. Specifically, a complete human-machine interaction should involve the following exchange of information: (1) the human worker, with the knowledge of the working scenario, actively provides input to the machine; (2) the machine, upon receiving the input instructions, proceeds to work on its dedicated duty; (3) after finishing its job, the machine returns output to the worker, notifying its availability; (4) the worker, knowing that the machine is once again available, provides it with further instructions. Based on this logic, the proposed module is developed to capture and understand the human-machine interactions of an operating manufacturing system by analyzing the exchange of input and output information. This system is critical since it utilizes powerful machine learning algorithms to enable a flexible and easily deployable solution to monitor and analyze human-machine interactions without heavily relying on data communication protocols such as Tera Term and MTConnect.

In typical scenarios, workers deliver input to the machines by performing operations on an interactive terminal. Such an operation may include pressing a button on the panel, entering commands via keyboard, etc. Similarly, the feedback of a machine is usually presented to the worker via text (or symbolic) format on the screen of the same panel. The proposed module consists of a wearable camera on the worker that captures the worker’s vision during a valid human-machine interaction to capture this kind of information flowing between the worker and the machine. The camera captures an RGB image $I_{HIM}$ and feeds it into the HIM. By detecting and filtering out the contours of the raw images, HIM can identify the panel’s position through the worker’s vision and utilizes text detection and recognition algorithms to extract the machine’s feedback to the user. Furthermore, the module identifies the position of the worker’s fingers over the panel’s buttons to acquire the operator’s input to the machine. The proposed module can function automatically and in real-time for the consistent supervision of human-machine interactions. After the internal processing, the HIM outputs a list $L^{\prime }$, each element is a command and its status. The command is the text from the control panel, and the worker’s gesture determines the status in $I_{HIM}$. The resulting $L^{\prime }$ is transferred to the state transition logic unit when this information is collected correctly. Together with *L*, the DMM and the current machine state generate the operating state information.

Because the computer processes each frame much faster than the worker can operate, most frames’ recognized instruction status in $L^{\prime }$ is idle. When the $L^{\prime }$ of an HIM returned in a frame contains a change of instruction status, the information is passed to the status logic unit, which will find the machine state change that the instruction may cause according to the current status. Such information is predefined by the user in the file system and can be called by the status logic unit. The new state will then be buffered in memory for five frames. After five frames, the buffered result is compared with the result from DMM and decides if the proposed next machine state is acceptable.

### 2.4. Hardware Requirement

Generally speaking, object detection relies on two different hardware systems: one is based on the generation of 3D point cloud information captured by LiDAR [18], which has the advantage of providing high-resolution information about the surroundings but comes with a high hardware cost, massive data storage, and computing requirements; the other, consumer RGB vision systems, have recently become very effective with regards to low cost and improved fidelity. In addition, the rapid development of AI techniques has dramatically improved object detection results for these systems. For these reasons, AIMS utilizes a computer vision model based on RGB images to obtain high precision results with the highest cost performance. Another important consideration is the choice of the computing model. Advanced computer vision models can use ordinary photos to obtain high accuracy with the rapid development of AI algorithms, and standard web cameras can obtain sufficient high-resolution images. Although it takes several hours for a moderately provisioned GPU to train a computer vision model, the amount of computation in making a prediction is manageable. Therefore, AIMS chooses to use the computing power provided by cloud services for model training. After the training, the computer vision model is deployed on an affordable embedded device. Specifically, the Vision Processing Unit (VPU) has been introduced by several Key players such as Nvidia, Intel, Google, Samsung, etc. VPUs are tiny portable devices specifically designed to handle visual models. With VPUs, developers can obtain sufficient real-time computer vision processing powers at a meager hardware cost. Overall, the hardware requirements of AIMS are only the web camera for image capture and the embedded device for prediction.

### 2.5. Evaluation of The Model

This model aims to monitor the machine’s state in real-time and detect machine failures in time. Faults include machine parts moving in a manner that does not conform to the current state, machine state transitions that do not conform to a predefined process, and machine states that do not conform to interaction expectations after worker interaction through the panel. Therefore, we defined the following criteria to measure the model’s performance.

After each frame is processed, the system compares the machine prediction state with the actual state. If they agree, the prediction is judged to be correct. Moreover, if the actual state is not ERROR, a correct prediction is considered a true positive, and an incorrect prediction is considered a false positive. If the actual state is ERROR, a correct prediction is considered a true negative, and an incorrect prediction is considered a false negative. Under such a criterion, *precision* reflects the model’s ability to detect machine errors, and *recall* reflects the model’s ability to track the normal working state of the machine.

## 3. Experiment: Case Study on a 3D Printer

In this section, we examine the feasibility and efficiency of the developed AIMS System in the use case of 3D printing. In this case, the general machine is the 3D printer.

### 3.1. Analysis of Supervised Production Equipment

**Figure 2:**
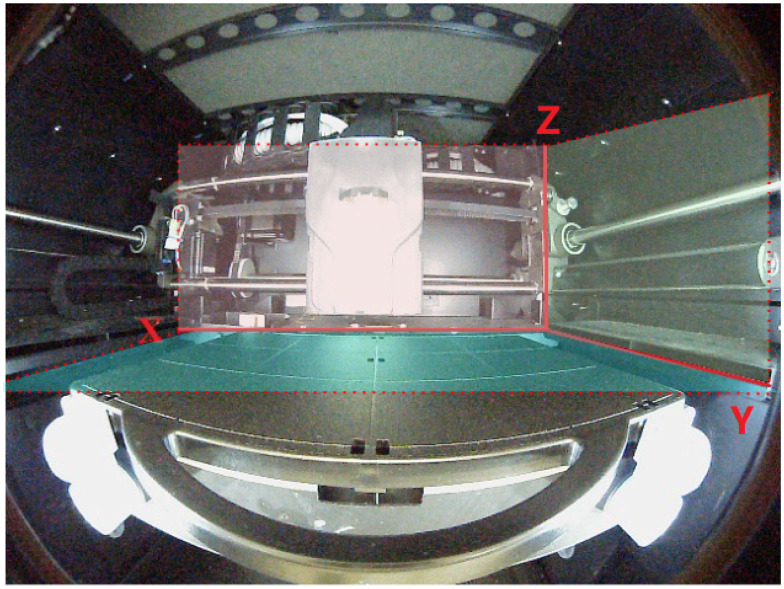
The canonical right-handed coordinate system.

The work of the 3D printer relies on the synergy of three components: the extruder, the build plate, and the motor axis. The extruder is filled with raw printing material. At the bottom of the extruder is the nozzle that constructs the printed item. The *canonical right-handed coordinate system* is considered, as illustrated in Figure 2, where the extruder is positioned in the plane restricted by the x and z axis, the build plate is in the plane restricted by the x and y-axis, the y and z-axis in the plane restrict the motor axis. Specifically, the x-coordinate of the print-head in the coordinate system can be obtained by looking at the extruder’s position. The build plate is the platform under the extruder that supports the printed object. The build plate can move in the vertical direction, so the z-coordinate (i.e., vertical position) of the print-head is collected by looking at the build plate. The motor axis is on the side of the extruder and can control the back and forth movement of the extruder. The nozzle’s y-coordinate (i.e., lateral position) is controlled from the coordinate system’s perspective. The extruder’s x-coordinate (i.e., horizontal position) can be directly obtained by analyzing the absolute position of the extruder. Consequently, by observing these three components, the AIMS can obtain indirect information about the motion of the nozzle. When this printer works, the print head motion changes significantly when a state transition occurs. With the information about the motion of the print head, our model can accurately sense the occurrence of state transitions. At each specific state, the nozzle moves in a specific way. Our model detects an error when an unexpected change in motion and the state transition are unsatisfied.

The printer was placed in a simulated factory environment in our lab. Under these conditions, the main thing that affects the computer vision model is the surrounding lighting conditions. The required conditions in the lab are relatively simple. There is one central lighting system inside the lab and one inside the printer. These two lighting systems may be on or off while the printer operates. Therefore, when training the model, the four possible lighting conditions are considered to ensure that our model makes accurate predictions under each of these conditions. The lighting conditions are also included as a factor during testing.

### 3.2. Building the Model for Direct Monitoring

The technique of artificial intelligence computer vision is leveraged in AIMS to automate the monitoring of the extruder, the build plate, and the motor axis. The AIMS AI computer vision system was configured to monitor the 3D printing system with main components consisting of the extruder, the build plate, and the motor axis to demonstrate model building. A camera was placed inside the 3D printer, ensuring the three main components were captured. The printer performed multiple print jobs with the camera to capture the images inside the printer at a rate of five frames per second. Each image is coded as a 3 (RGB channels) by *H* by *W* tensor to represent the colours of each pixel of the image. The image contains *H* times *W* pixel for each channel and can be regarded as an *H* by *W* matrix. The most common and intuitive way to represent an object in pixelated images is to use a bounding box, which can be understood as a rectangle within which the object is placed. Mathematically the coordinates of the center of the rectangle on the *H* by *W* matrix can be used to determine the unique corresponding bounding box. After enough images are collected, the bounding boxes of the extruder, build plate, and motor axis in each image with the corresponding type of bounding box are labelled, which is the final step in data preparation for model training.

**Figure 3:**
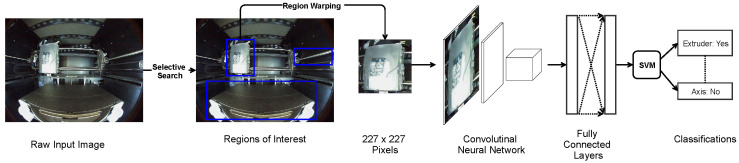
High-level architecture of the Region-based Convolutional Neural Network.

The state-of-the-art methods for object detection are deep learning models based on convolutional neural networks (CNN), such as Region-based Convolutional Neural Networks (R-CNNs) [19], and Single Shot object Detectors (SSDs) [20]. As illustrated in Figure 3, R-CNN first segments the input image into small regions and then combines them greedily using selective search based on similarity in certain features. For each combined region (i.e., a region of interest), R-CNN warps it to a 227 × 227 (pixels) fixed-size image and delivers it through a CNN for feature extraction at different levels of precision. The extracted features are passed through fully connected neural network layers for compilation and support vector machines (SVMs) for classification. Combining selective regional search with the traditional CNN models allows the network to handle input images that consist of compact objects with distinct features. R-CNN generally achieves greater accuracy in object detection and instance segmentation through the region-based searching method. However, its slow processing speed renders it less effective in real-time analytic scenarios. Predominant object detection models based on the R-CNN architecture include Fast-RCNN [21], and Mask-RCNN [22].

Also of interest are Single Shot Detectors (SSD) which consist of a backbone neural network for extracting features and convolutional layers for making predictions. Unlike R-CNN, SSD predicts both the classifications of objects and the bounding box locations simultaneously at convolutional filtering kernels. More specifically, input images fed to the SSD require predetermined ground truths bounding boxes and class labels before training. Subsequently, the image is separated into regions containing anchor bounding boxes predefined in shape and serve as initial guesses. The input image is encoded and passed through a pre-trained feature extractor, such as the Visual Geometry Group from Oxford (VGG16) [23], to obtain feature maps. By maximizing the Intersection over Union between anchor bounding boxes and the ground truths, the detector identifies the positive samples for the learning framework. Finally, convolutional layers with decreasing scales produce classification results for objects of various sizes in the input image. Compared to R-CNN, SSD achieves greater inference speed during testing due to its predefined anchor bounding boxes that effectively eliminate the time required for regional proposals. Therefore, despite SSD’s detection accuracy seldom outperforming R-CNN, its competent speed makes it especially suitable for real-time object detection scenarios. Predominant object detection models based on the SSD architecture include the YOLO family.

For the construction of the direct monitoring model, several cutting-edge models were selected, trained, and compared in three dimensions to find the most suitable model to handle the internal conditions of the 3D printer. Specifically, YOLOv3 [24], YOLOv4 [25], and Mask-RCNN were selected for testing. The two evaluating criteria are the performance in the standard COCO dataset [26] and the dataset produced in this project describing the internal condition of the printer. The performance is measured by mean Average Precision [27] at intersection over union [28] equals fifty percent (mAP 50). To ensure that the AIMS can promptly detect changes in the machine state even with the limited computational power of the embedded device in real production, the processing frequency of each model is also included as an important indicator. The final test results are shown in Table 1.

Based on the tests, the accuracy of YOLOv4 has a clear advantage over the standard COCO test. However, this advantage was not evident in our 3D printer dataset. Also, the research team observed that YOLOv3 has a higher processing speed in the 3D printer than the other models. To best accommodate the use case of the 3D printer, the convolutional computing-based model YOLOv3 is adapted. YOLOv3 uses up to 53 convolutional layers, reducing the computational speed but improving prediction accuracy. Furthermore, when used in the AIMS, the computational speed of YOLOv3 is sufficient to complete the system’s work requirements. YOLOv3 obtains the final prediction through a multi-layer feature extractor. The first layer obtains the basic feature after multiple convolution calculations and then combines it with the feature of the previous unsampled data into the next extractor layer. In the last layer, it goes through a similar process again. With this three-layer design, the model can obtain both the original data and the features extracted from intermediate calculations to obtain more accurate predictions. When using this model, the prediction of the bounding box is made from three scales: (1) the position of the bounding box, (2) the confidence of the object present at that position, (3) and the class of the object labeled by the bounding box.

### 3.3. Building the Model for Human-Machine Interaction Monitoring

**Figure 4:**
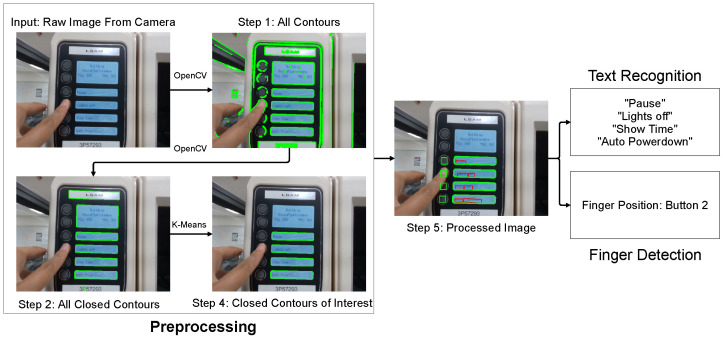
Workflow of the finger-text detection module.

**Preprocessing.** Before feeding the raw images captured by the worker’s headset camera into the subsequent machine learning models, preprocessing is required to ignore unnecessary background details and to narrow down the regions of interest (e.g., the interactive display screens and the push-buttons) to achieve optimal efficiency, as illustrated in Figure 4. Utilizing OpenCV’s [29] functionalities of calculating the area and perimeter of detected contours, the preprocessing module first identifies all the closed contours within the input raw image. Then, by feeding the sizes of all the closed contours into a K Means Clustering algorithm [30], the module filters out all the closed contours with a similar size to the customized region of interest. In an arbitrary working condition, this preprocessing step can be taken to enhance the robustness of the model by discarding distracting information and focusing on the preset regions of interest. In the case study of a 3D printer, the regions of interest that the preprocessing step filters out and focuses on are the text display screens on the right of the control panel.

**Text Detection and Recognition.** After locating the display screens of the machine, the module performs text detection and recognition on the filtered regions to extract the machine’s output, which is illustrated in Figure 4 with a sample frame. Character Region Awareness for Text Detection (CRAFT) [31] is a novel text detector that, when given an input image, first localizes individual characters by producing region scores that indicate the probability of a given pixel being at the center of a character. After localizing the characters, CRAFT’s prediction groups adjacent characters into instances by calculating the affinity scores for each gap between characters, which reflect the probability of adjacent characters being in the same word. Since CRAFT detects individual characters before making predictions on words, it can locate the positions and boundaries of deformed texts, such as those with curved orientations. Considering that the images captured through the vision of workers during human-machine interactions usually include distortions due to perspective projections, CRAFT’s high flexibility in detecting texts of complicated shapes and sizes allows it to be applied to dynamic manufacturing conditions. Furthermore, CRAFT’s inference step of producing bounding boxes around detected text instances is defined by producing enclosing rectangles at a pixel level. CRAFT achieves competent speed during inference without further post-processing (such as searching for relations between text components). It is, therefore, suitable for text detection in real-time. Therefore, the preprocessed raw images from the worker’s wearable camera are fed into the CRAFT model in this case study. The positions of the displayed texts on the region of interest (printer panel) are highlighted with bounding boxes. The bounding-box outputs are delivered through a unified four-stage scene text recognition algorithm developed by Jeonghun Baek et al. [32], which translates the highlighted pixels into corresponding text strings. The preprocessing step of narrowing down the regions of interest and CRAFT, which enables accurate detection of distorted texts, allows the proposed module to function in diverse working conditions efficiently.

**Figure 5:**
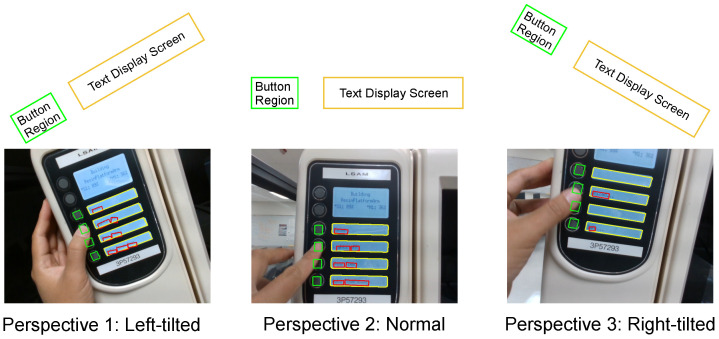
Processed camera frames from different visual perspectives.

**Locating Push Buttons.** To monitor the human’s input to the machine, the module first detects the locations of the push buttons that the human interacts with. Since the buttons may be of various shapes and sizes in diverse working scenarios, the proposed model predicts the position of the buttons via specific calculations rather than depending on the OpenCV’s functionalities. In this case study of the 3D printer, there is a push button to the left of each text display screen. Therefore, after identifying the contour coordinates of the display screens in the previous preprocessing steps, the module calculates the relative positions of the pushbuttons. Since the wearable camera is located on the headset of the human operator, the image frames captured will inevitably contain certain distortions and tilt. Therefore, the algorithm for locating the button regions should accommodate different perspectives of the human operator’s vision, as illustrated in Figure 5.

**Figure 6:**
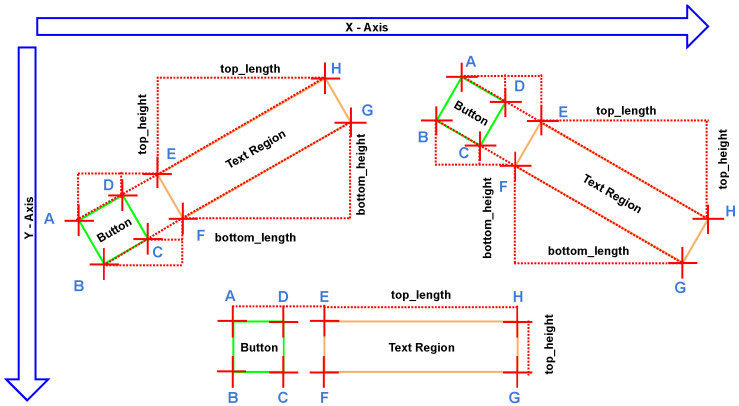
Illustration of determining button positions of different visual perspectives.

Since the positions and sizes of the buttons relative to those of the text regions do not change when there is a transformation of the camera’s perspective and angle, the algorithm can identify the regions of the buttons by extrapolating the contour coordinates of the text regions following the perspective transformation, as illustrated in Figure 6.

For each scenario, the *x*-coordinate (horizontal coordinate) and the *y* coordinate (vertical coordinate) of every corner vertex need to be calculated to determine and draw the bounding boxes of the button regions. The ratios of lengths are defined as:

(1) $$\frac{AE}{EH}=\alpha \;,\frac{DE}{EH}=\beta \;,\frac{BF}{FG}=\gamma \;,\frac{CF}{FG}=\delta$$

Due to the properties of similar triangles, these ratios of lengths will not change dramatically during the perspective transformation.

For the first scenario in which the camera angle is horizontal, no specific transformation is required since the corner coordinates of the button regions and the text regions are aligned horizontally and vertically. The length of the text region contour can be calculated as

(2) $$top_{length}=H_{x}-E_{x}$$

Therefore, the (x,y) coordinates of the vertices of the button region can be calculated as follows (note that the (x,y) coordinates for the vertices E,F,G,H are known from the preprocessing steps by identifying the contours of the text regions):

(3) $$\begin{array}{cc} A_{x} & =B_{x}=E_{x}-\alpha \times \left(top_{length}\right)=E_{x}-\alpha \times \left(H_{x}-E_{x}\right)\\ D_{x} & =C_{x}=E_{x}-\beta \times \left(top_{length}\right)=E_{x}-\beta \times \left(H_{x}-E_{x}\right)\\ A_{y} & =D_{y}=E_{y}=H_{y}\\ B_{y} & =C_{y}=F_{y}=G_{y} \end{array}$$

For the second scenario in which the camera angle is left-tilted, the coordinates of the button vertices are no longer at the same horizontal and vertical angle. Since there is a shift in vertices’ positions, the lengths and heights’ calculations need to be changed accordingly. The top lengths and heights of the text region contours are defined as: $top_{length}=H_{x}-E_{x}$, $top_{height}=E_{y}-H_{y}$. Similarly, the bottom lengths and heights of the text region contours can be defined as: $bottom_{length}=G_{x}-F_{x}$, $bottom_{height}=F_{y}-G_{y}$. Even though, in theory, the bottom lengths and heights should be the same as those of the top, separated calculation and treatment are expected to further enhance the robustness of the module, especially in cases where the contour vertices are not detected by the preprocessing steps precisely. Therefore, the (x,y) coordinates of the vertices of the button region can be calculated as follows:

(4) $$\begin{array}{cc} A_{x} & =E_{x}-\alpha \times \left(top_{length}\right)=E_{x}-\alpha \times \left(H_{x}-E_{x}\right)\\ A_{y} & =E_{y}+\alpha \times \left(top_{height}\right)=E_{y}+\alpha \times \left(E_{y}-H_{y}\right)\\ B_{x} & =F_{x}-\gamma \times \left(bottom_{length}\right)=F_{x}-\gamma \times \left(G_{x}-F_{x}\right)\\ B_{y} & =F_{y}+\gamma \times \left(bottom_{height}\right)=F_{y}+\gamma \times \left(F_{y}-G_{y}\right)\\ C_{x} & =F_{x}-\delta \times \left(bottom_{length}\right)=F_{x}-\delta \times \left(G_{x}-F_{x}\right)\\ C_{y} & =F_{y}+\delta \times \left(bottom_{height}\right)=F_{y}+\delta \times \left(F_{y}-G_{y}\right)\\ D_{x} & =E_{x}-\beta \times \left(top_{length}\right)=E_{x}-\beta \times \left(H_{x}-E_{x}\right)\\ D_{y} & =E_{y}+\beta \times \left(top_{height}\right)=E_{y}+\beta \times \left(E_{y}-H_{y}\right) \end{array}$$

For the third scenario where the camera angle is right-tilted, a similar transformation of coordinates can be applied to obtain the relative positions of the button regions on the image frames. By feeding the (x,y) coordinates of the four button region vertices to the OpenCV’s functionality of plotting polygons, bounding boxes can be drawn that remain accurate regardless of the possible shift in angles of the wearable camera.

**Finger Detection.** After locating the press-button regions, the interactions and inputs from the human operator can be captured by detecting whether the finger is present above certain buttons. Instead of adopting finger detection models implemented with computer vision techniques, the module utilizes OpenCV’s extract pixel color values from frames. Specifically, with the bounding box regions (i.e., the vertex coordinates of the button regions) calculated in the previous step, the module extracts the RGB color values for each pixel within the button regions. By calculating the averaged RGB values for each button, the module identifies which button the human operator interacts with by picking out the button position with the most distinct averaged RGB values. To deal with edge cases in which the human operator’s finger covered more than one button, the module always prioritizes the button position with the least *x* coordinate value (i.e., to the top of the image frame), where the fingertip is assumed to be located at. In other possible cases, when the text-display regions are occluded (which happens most often when the right hand is used to operate the panel), the areas blocked for more than 50% area are omitted. Since in most human-machine interaction scenarios, the fingers other than the index finger are curled up, the occlusion of text regions by the hand and fist should not interfere with the button regions of interest that the index finger is positioned at. Therefore, these methods detect the left-hand and the mirrored right-hand interactions with the panel press buttons. Consequently, this module only focuses on the interactive button regions of interest. It can thus achieve greater accuracy than machine learning models that try to identify and predict the whole finger globally. Therefore, even though this implementation of finger detection is less sophisticated than those that adopt computer vision models, its simplicity and efficiency are most suitable for the real-time monitoring of human-machine interactions.

## 4. Results

### 4.1. Experimental Setup

Before the DMM training, the 3D printer printed two items. At the same time, a camera inside the machine recorded the entire working process. During this process, the experiments continuously switched between four possible lighting conditions. The final 1500 images were put into the training set, with about a quarter of the overall images from various working conditions. The research team then annotated each image with the extruder, build plate, and axis with the bounding box.

### 4.2. DMM Result

The cloud computing approach was used for the training of the model. The labeled 1500 training images were put on the Colab server [33] provided by Google, and the NVIDIA T4 assigned by Colab was trained with 100 epochs. With this configuration, each epoch took an average of 300.198 s to complete. The whole training took about 250 min to complete. The trained model was immediately tested on the server. The DMM reached a data processing rate of 137 images per second on this server with 99.98% mean Average Precision at the intersection over union equals fifty percent.

**Figure 7:**
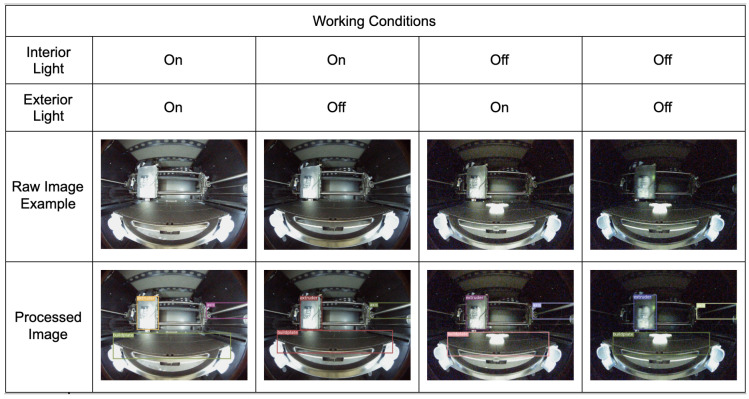
Some results on different working conditions.

The model was then deployed to the low-cost solution VPU NVIDIA Jetson Nano [34] with 128 Cuda computation units [35] and Jetson Xavier NX [36] with 384 computation units. Then the model is evaluated on a pre-prepared test dataset of 216 frames. Figure 7 shows some results from different working conditions. From the figure, it can be observed that the model recognizes the extruder, the build plate, and the axis accurately.

When the model finishes processing an image, it generates a predicted bounding box that encloses some pixels on the image, and we use $P_{p}$ to denote the set of these pixels. We also use $P_{g}$ to represent the set of pixels inside the ground truth bounding box. After that, we calculate intersection over union (IOU) as

(5) $$IOU=\frac{\left|P_{p}\cap P_{g}\right|}{\left|P_{p}\cup P_{g}\right|}\in \left[0,1\right]$$

A higher IOU represents a higher degree of overlap between prediction and ground truth. IOU is used as a criterion to distinguish true positives, false positives, and false negatives by comparison with a given threshold. When a prediction is generated, its IOU is calculated and compared with a standard. The prediction is a true positive if the computed IOU is higher than the predefined standard. Otherwise, the prediction is a false positive. It is considered a false negative if there is a ground truth bounding box without any prediction.

For a given IOU standard, a recall-precision curve [37] can be calculated by computing the recall and precision when including predicted bounding boxes one by one in decreasing orders of their prediction confidences. For a specific class of object, the average precision is defined as

(6) $$AP=\sum_{i=1}^{n}P_{i}\left(R_{i}-R_{i-1}\right),R_{0}=0$$

where $P_{i}$, $R_{i}$ is precision and recall when the most *i* confident predictions are included, and *n* is the number of predicted bounding boxes. The calculated AP is approximately the area under the recall precision curve.

The numerical results in the average precision calculated with a IOU standard of 0.5 for each component in all conditions are listed in Table 2. The overall mean average precision is 0.99807. When plugged into the camera and processing the data in real-time, the Jetson Nano averaged 2.3 frames per second (FPS), and Jetson Xavier averaged 16.2 FPS. This processing speed was below our expectations. However, the processing speed of Xavier is sufficient to maintain a decent accuracy for the 3D printer. Under more complex working conditions, such a speed might cause poor performance of AIMS, and consequently, more lightweight models such as Mobilenet ([38]) should be tested to improve the speed of processing.

### 4.3. HIM Result

**Figure 8:**
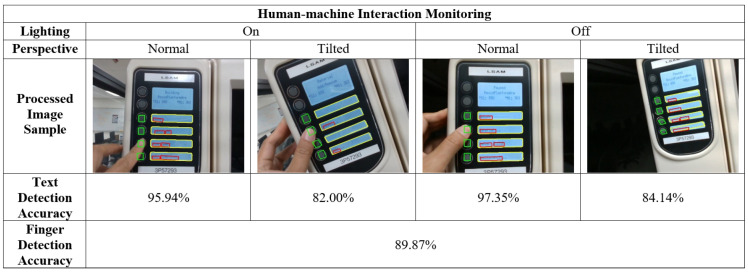
Test results for the Human-machine Interaction Monitoring.

The human-machine interaction monitoring module test images are collected from the wearable camera on the operator’s headset and during the same process described in Section 4.1. Specifically, 1500 image frames with valid human-machine interactions and different lighting and perspective settings are selected as the test set. After performing the HIM module on the entire test set, the results are summarized in Figure 8. Note that a text detection prediction was considered accurate if all the four text regions were predicted the same as the ground truths. Similarly, a finger detection prediction was deemed accurate if all four press-button regions’ positions were correctly predicted compared to the ground truths.

The test accuracy for the text detection model is optimal for the setting in which the exterior lighting is dark, and the wearable camera is not tilted. The high contrast in the lighting of this scenario allows better detection of the display screen contours. At the same time, the leveled perspective rules out further complications in calculating press-button contours. The text detection model can achieve competent accuracy and robustness in all the test environments set for the experiment.

The finger detection model, utilizing the method of color comparison of bounding-box regions, depends on the accuracy of the previous model’s press-button detection. In the 1500 total test frames, 1348 frames accurately identified all the press-button regions compared with the ground truths, achieving an average accuracy of approximately 89.87%. For all the correctly identified press-button regions, the finger detection model achieved 100% perfect accuracy in detecting which press button the operator’s finger is interacting with. Therefore, the entire HIM module’s performance mainly depends on the text detection model’s accuracy.

### 4.4. AIMS Result

The AIMS system was deployed on the 3D printer Stratasys uPrint in the lab. All the information tracked by the AIMS was transmitted to the graphic user interface in real-time through the LAN, making it easy for the staff to monitor the working status of the machine remotely when needed. Two tests were conducted to evaluate the effectiveness of the AIMS system.

#### 4.4.1. Normal Operation Test

**Figure 9:**
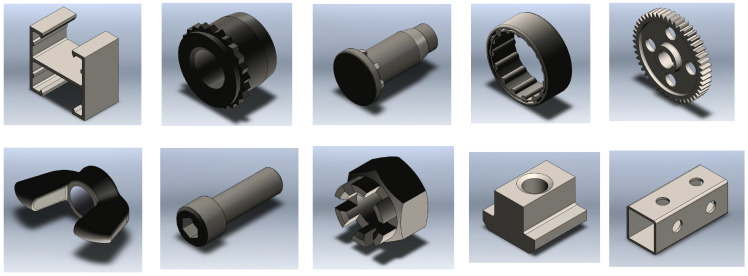
The set of items used for the normal operation and the abnormal condition tests.

The first one was the machine’s regular operation test. In this test, we used the 3D printer to create ten items under various lighting conditions. Figure 9 illustrates the example items used for the tests. These ten different tasks were designed to cover all possible legitimate workflows. In half of the tests, an operator wearing the HIM monitoring system operated the control panel in the lab to confirm that the HIM was accurately monitoring the operator’s commands and passing the collected information to the Machine State Management Logic Unit of AIMS.

AIMS has outstanding performance in regular machine operation. The AIMS followed the whole machine operation process in all nine tasks, including five times when a worker operated the panel. Afterwards, the 3D printer was adjusted to its initial state and was tested against the failed items again. This time the AIMS completed the job accurately.

Within all frames of these ten tasks, we found that the model gave incorrect predictions or failed to detect the object in rare cases, three frames to be specific. However, since the AIMS system uses a boolean First-in-First-out queue buffer of up to five frames to determine if there is a fault or state change, as long as more than 80% of the model predictions are accurate, the AIMS system will work accurately. For example, if the model predicts correctly in the previous frames, and suddenly the lighting condition has changed, or something briefly blocks the camera’s vision. This might result in the misprediction of two or three frames. However, the condition returns for the subsequent frames normal; thus, the prediction continues to work as expected. In this case, these few frames of misprediction, not exceeding five consecutive frames, will not be considered as a change in the state of the entire machine but as an accidental mistake and will not influence the prediction of the entire machine. As a result, these few prediction errors did not cause the failure of AIMS.

#### 4.4.2. Abnormal Condition Test

The second test is the machine abnormal condition test. In this test, we also used the 3D printer to create ten items under various lighting conditions. However, on the way to the printer’s task, the research team intervened several times to make the printer jump out of the pre-defined standard workflow. This was used to test whether the AIMS system could detect abnormalities in the machine state. AIMS also performed very well in the machine anomaly monitoring test. 3D printers were successfully monitored for state anomalies within five frames after artificially created anomalies in 9 out of 10 jobs. However, in 1 task, the research team stopped the 3D printer at Calibration, a state of readiness before the actual print job began. The team named this test as halt test. In actual production, such a state would be equivalent to a machine whose workflow is blocked in a particular state for some reason, thus causing production to stall. However, since the machine components are stuck in their legitimate range of motion in their current state when this fault occurs, AIMS’s Abnormal Checker does not consider this a fault. In the next step, a reasonable solution is to include a heuristic time estimate for each machine state when defining its workflow. If AIMS finds a state severely overrun, it issues a warning.

#### 4.4.3. Numerical Result

For each frame during the test, the prediction results of AIMS are evaluated based on the scheme defined in Section 2.5, where each prediction is classified as one of true positive, false positive, true negative, and false negative. After all the tests are down, the results of 19 tests are put together to compute overall accuracy, precision, and recall. The data from the halt were omitted because the machine stopped at a specific state and generated many repeating data. Again, with the criterion defined in Section 2.5, precision reflects the model’s ability to detect machine errors, and recall reflects the model’s ability to track the normal working state of the machine. The results are listed in Table 3.

The data shows that the model performs well in tracking the regular operation and detecting the fault. By taking a closer look at the prediction results, the team finds that most false negatives and positives are mainly located around the machine’s state transition. A 5-frame buffer must be filled before AIMS issues a state transition when the machine changes its state.

## 5. Discussion

### 5.1. Application

The proposed method is **ASAP** (Affordable, Scalable, Accessible, Portable) during application.

Specifically, it is **affordable** as all the hardware used in the proposed method is cost-effective compared with existing solutions. The requirement of image quality of the algorithm is not high, ordinary camera sensor units can be used instead of the ones with high specs, and thus is affordable. Running the algorithm for detection also does not require high computational power or expensive commercial software. The proposed method can work without a certain protocol and thus avoid the expensive installation fees of such systems. For some machine models, there exist some protocols that can access the machine log and get the machine state. However, it is common for different manufacturers to use different protocols for their machines, and a factory might use various brands of machines. In this case, some commercial software or certain hardware (data loggers, protocols)might be needed to monitor all the machines of interest, and installing them might account for a sizable portion of the budget. In the proposed algorithm, this challenge could be solved as it is compatible with the existing portals as a bonus but does not require any protocol or the installation of additional hardware systems.

The proposed method is **scalable** as it could be expanded and scaled up if the factory already has other existing sensors or IoT devices such as thermal cameras and smart meters to extend its functionality or increase its accuracy. With more sensors, the information the system can collect will significantly increase. By editing the proposed algorithm to make it compatible with the additional information and properly using it, the system can be extended in terms of accuracy and functionality. For example, based on the model’s prediction, a virtual tutorial can be easily added as its functionality, which can give step-by-step instruction to workers that are new to the operation of machines and detect misoperation to reduce the risk of operation. This algorithm can also be used for machines other than the 3D printer with minimal code editing and potentially on any machine where a camera can capture the human-machine interaction process. The relevant work has been published, where the proposed method is scaled to the more convoluted real-time monitoring of operators’ interactions with an assembly line [39].

The proposed method is **accessible** as the framework is automated and fully encapsulated. It can be used by workers without advanced knowledge of computer vision or understanding of the protocols. Other methods of monitoring the machine state might require the workers to get familiar with the protocols of machines. If a factory uses machines from different manufacturers, each worker has to learn multiple kinds of protocols and needs to be very familiar with the machine log from each machine to keep track of the machine’s state. The proposed method does not require such knowledge. As long as the model is trained, the prediction process can be done automatically, and the prediction result will be shared with the workers in a very trivial way. Even in cases of using various machines, only minimal changes to the model need to be made. Once the model has been modified and trained by engineers, no additional effort needs to be made to understand the usage of the proposed system on different machines.

The proposed method is **portable** as the data is collected from a portable camera, does not require a very high resolution for the prediction, and is processed by the portable NVIDIA Jetson kit. The proposed method can work perfectly in portable conditions by using portable hardware and lightweight visual data, which is fast for wireless data transmission. All the hardware involved in the detection process can be portable. For example, the camera can be installed on a pair of goggles or a helmet, which connects to a microcomputer system such as a Raspberry Pi.

### 5.2. Limitations

The proposed algorithm has two major limitations. The algorithm is not super robust for handling extreme cases when consecutive frames following a distribution not seen in the training sample were captured by the camera. All the existing machine learning-based object detection algorithms assume that the images will be processed following the same distribution as the training data. When the above assumption fails, an unexpected result may be generated. Consequently, any machine state monitoring algorithm that relies on object detection may fail due to image distribution shifting caused by unstable light conditions, unexpected blocking obstacles, or unusual vibration. As mentioned in Section 2.1.2, a FIFO transition method which allows an error tolerance of around twenty percent and increases the robustness of the detection does exist in the proposed method, but in extreme cases, the algorithms might still produce misprediction.

The second major limitation is that DMM (3D printer interior object detection using YOLO) requires a lot of training samples with manual labeling of ground truths bounding boxes, a common unavoidable problem in the computer vision field. The data collection, labeling and training of the machine learning model may cost some human labor and time when the system gets set up before it can be used effortlessly with trained models.

### 5.3. Future Work

In the future, more use cases (other than 3D printing and assembly lines [39]) with different kinds of interfaces and human-machine interactions might be considered to expand the generality of the proposed method. In terms of the functionalities, interactive Augmented Reality (AR) training will be developed upon the proposed framework by leveraging the finger detection and text recognition algorithm through the AR goggles.

## 6. Conclusions

This research proposed the ASAP solution to automate the supervision in manufacturing and significantly reduce the cost of smart manufacturing deployment in SMMs. In contrast, the cost of training AI models has decreased rapidly due to the development of cloud computing technology in recent years. This study developed the AI-assisted Machine Supervision (AIMS) system that provides SMMs with two major subsystems: direct machine monitoring (DMM) and human-machine interaction monitoring (HIM). The experimental results of a case study in 3D printing validated the feasibility of the AIMS system with affordable VPU configuration and evaluated the efficiency of machine supervision for an ASAP solution.

The AIMS system will impact smart manufacturing workers by empowering them with actionable intelligence to contribute to decision-making for machine operation management, production scheduling, demand-side facility management for reducing operation cost, and improved productivity for healthy, safe, and accessible manufacturing environments in SMMs. Essentially, the ASAP AIMS system will function as a human-machine system to engage people and systems in complex data management and human-centered workflow automation and control.

## Figures and Tables

**Table 1:** The performance and frequency of three candidate models, where AP stands for mean average precision when IOU defined in Equation (5) are 0.5, and FPS stands for frame per second, the processing rate.

	COCO		3D Printer	
	AP	FPS	AP	FPS
YOLOv3	51.5	23.8	98.48	29.4
YOLOv4	64.9	19.2	99.80	22.3
Mask-RCNN	60.0	5.00	98.80	5.90

**Table 2:** The accuracy of DMM on the test dataset, where AP is average precision.

Index	Class Name	AP
0	extruder	0.99935
1	buildplate	0.99946
2	axis	0.99539

**Table 3:** The numerical result of 19 tests for AIMS.

Precision	Recall
0.923	0.936

## Data Availability

Not applicable.
